# Multi-perspective comparison of the immune microenvironment of primary colorectal cancer and liver metastases

**DOI:** 10.1186/s12967-022-03667-2

**Published:** 2022-10-04

**Authors:** Yangsong He, Yanan Han, A-hui Fan, Danxiu Li, Boda Wang, Kun Ji, Xin Wang, Xiaodi Zhao, Yuanyuan Lu

**Affiliations:** 1 Department of Gastroenterology, Tangdu Hospital, Fourth Military Medical University, Xi’an, 710038 China; 2 State Key Laboratory of Cancer Biology and National Clinical Research Center for Digestive Diseases, Xijing Hospital of Digestive Diseases, Fourth Military Medical University, Xi’an, 710032 Shaanxi China

**Keywords:** Colorectal cancer, Liver metastasis, Immune microenvironment, Immunosuppression, Comparison

## Abstract

**Background:**

Liver metastases are a major contributor to the poor immunotherapy response in colorectal cancer patients. However, the distinctions in the immune microenvironment between primary tumors and liver metastases are poorly characterized. The goal of this study was to compare the expression profile of multiple immune cells to further analyze the similarities and differences between the microenvironments of liver metastases and the primary tumor.

**Methods:**

Tissues from 17 patients with colorectal cancer who underwent resection of primary and liver metastases was analyzed using multispectral immunofluorescence. The expression of multiple immune cells (CD8, Foxp3, CD68, CD163, CD20, CD11c, CD66b, CD56, PD-L1, INF-γ, Ki67 and VEGFR-2) in the tumor center (TC), tumor invasive front (< 150 µm from the tumor center, TF) and peritumoral region (≥ 150 µm from the tumor center, PT) was evaluated via comparison. The expression of CD68 and CD163 in different regions was further analyzed based on the cell colocalization method. In addition, different immune phenotypes were studied and compared according to the degree of CD8 infiltration.

**Results:**

The expression trends of 12 markers in the TF and TC regions were basically the same in the primary tumor and liver metastasis lesions. However, in comparison of the TF and PT regions, the expression trends were not identical between primary and liver metastases, especially CD163, which was more highly expressed in the PT region relative to the TF region. In the contrast of different space distribution, the expression of CD163 was higher in liver metastases than in the primary foci. Further analysis of CD68 and CD163 via colocalization revealed that the distribution of macrophages in liver metastases was significantly different from that in the primary foci, with CD68^−^CD163^+^ macrophages predominating in liver metastases. In addition, among the three immunophenotypes, CD163 expression was highest in the immune rejection phenotype.

**Conclusions:**

The immune cells found in the primary tumors of colorectal cancer differed from those in liver metastases in terms of their spatial distribution. More immunosuppressive cells were present in the liver metastases, with the most pronounced differential distribution found for macrophages. CD68^−^CD163^+^ macrophages may be associated with intrahepatic immunosuppression and weak immunotherapeutic effects.

**Supplementary Information:**

The online version contains supplementary material available at 10.1186/s12967-022-03667-2.

## Introduction

The incidence and mortality of colorectal cancer (CRC) rank third and second in the world, respectively [1]. A recent epidemiological report in China pointed out that the detection rate of stage IV colorectal cancer has increased, and the mortality rate has also increased [2]. Tumor metastasis is the main cause of death in these patients, and the most common site of metastasis is the liver. Surgery is currently the best treatment for liver metastases, and its 5-year survival rate can reach 20–45% [3]. However, resectability depends on many factors, including the extent of liver metastases, and whether there are other unresectable extrahepatic diseases and patient complications [4]. Therefore, for unresectable patients, effective treatments are urgently needed, and immunotherapy has become a hot spot of research. Studies have shown that immune checkpoint inhibitors have sustained clinical responses in colorectal cancer patients with high microsatellite instability (MSI-H) or mismatch repair deficiency (MMR-D), with significant clinical improvements reported [5, 6]. However, immunotherapy has shown a weakened therapeutic effect on liver metastases [7].The immunosuppressive microenvironment of the liver may be the reason for the weakened immunotherapy effect. In particular, studying the difference in the tumor microenvironment between primary colorectal cancer and liver metastases is necessary to understand the differential responsiveness to immunotherapy.

The tumor microenvironment is a complex and dynamic system that contains a variety of immune cells. Tumor-infiltrating T lymphocytes are regarded as the main effectors of anti-tumor immune response [8, 9], and CD8 is recognized as the important marker of T-cell infiltration. Foxp3 is an important marker of regulatory T cells. In different types of tumors, a large amount of Treg cell infiltration is associated with poor clinical prognosis [10], but its role in colon cancer is controversial [11]. CD68- and CD163-labeled tumor-associated macrophages (TAMs) have both tumor-promoting and antitumor effects on tumors [12, 13], and they both have significant correlation with prognosis. For example, a high CD163^+^/CD68^+^ ratio in the infiltrative margin of the tumor suggests a poor prognosis for the colorectal cancer patients. [14]. We also include CD20-labeled B lymphocytes [15], CD66b-labeled neutrophils [16], CD11c-labeled dendritic cells (DCs) [17] and CD56-labeled natural killer (NK) cells [18], which all play an important roles in the occurrence and development of tumors. In addition, PD-L1 is an important marker for the evaluation of immunotherapy [19], VEGFR-2 is one of the key markers of tumor angiogenesis [20], INF-γ is an important proinflammatory factor that inhibits tumor growth [21], and Ki67 evaluates cell proliferation and is also related to tumor prognosis [22]. In this study, we used these biomarkers to evaluate the overall tumor microenvironment of primary colorectal cancer and liver metastases from multiple perspectives to gain a deeper understanding of the differences in the tumor microenvironment.

## Materials and methods

### Patients and tissue samples

Seventeen paired primary CRC tissues and corresponding hepatic metastatic tissues were collected from patients who underwent concurrent resection from 2012 to 2020 at Xijing Hospital of Digestive Diseases, the Fourth Military Medical University. Those who received preoperative therapy, such as chemotherapy and radiotherapy, were excluded. The pathology is used to confirm the liver masses as metastatic CRC. This study was approved by Xijing Hospital’s Protection of Human Subjects Committee. All patients recruited in this study were informed before participating, and their clinicopathological characteristics were summarized in Table 1.

**Table 1:** Clinicopathological characteristics of the patients

Clinic-pathologic features	Number (%)
Age	
Age (mean ± SD)	52.88 ± 14.44
Gender	
M le	9 (52.9)
Female	8 (47.1)
Location of the primary tumor	
Colon	10 (58.8)
Rectum	7 (41.2)
Number of liver metastatic lesions	
1	10 (58.8)
2	3 (17.6)
≥ 3	4 (23.6)
Maximum size of the metastatic tumor (cm)	
Median (range)	3.0 (0.5–9)
AJCC T stage	
T3	10 (58.8)
T4	7 (41.2)
AJCC N stage	
N0	2 (11.8)
N1	6 (35.3)
N2	9 (52.9)

### Multiple immunofluorescence staining

**Fig. 1:**
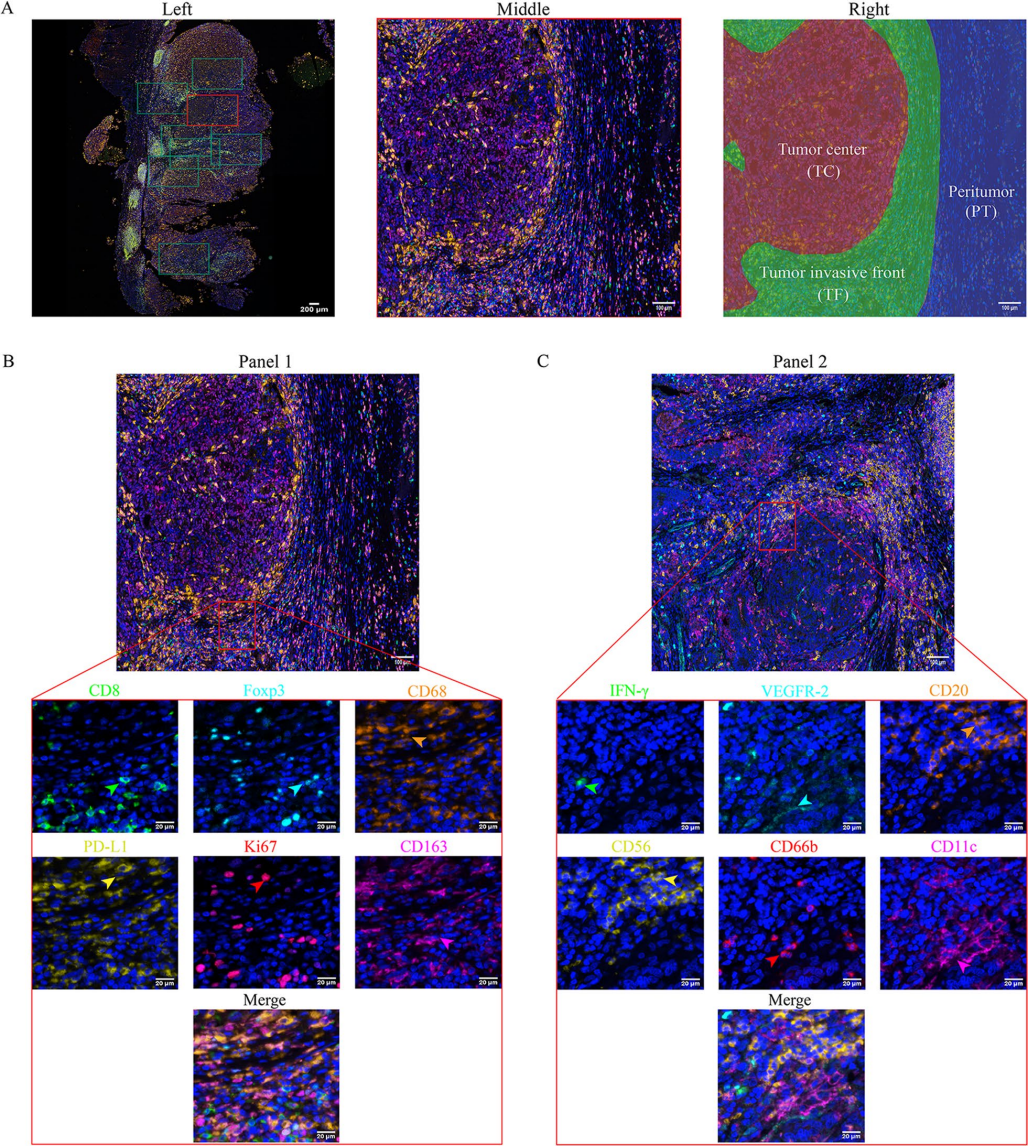
Image acquisition, partitioning and staining scheme. **A** left side: 10× scan of the whole film; middle side: 20× local area magnification imaging; right side: tumor center, tumor invasive front (less than 150 µm from the tumor) and peritumor (more than 150 µm from the tumor); **B** Staining Panel l (the green, cyan, orange, yellow, red and magenta arrows indicate positive cells with the expression of CD8, Foxp3, CD68, PD-L1, Ki67 and CD163 proteins in tumor tissue); **C** Staining Panel 2 (the green, cyan, orange, yellow, red and magenta arrows indicate positive cells with the expression of INF-γ, VEGFR-2, CD20, CD56,CD66b and CD11c proteins in tumor tissue)

The tissue samples stored in formalin were dehydrated and embedded in paraffin in accordance with conventional methods. Paraffin blocks were cut into 4–5 µm thick sections and mounted onto glass slides. Then, according to the manufacturer's instructions, the Opal 7-color IHC Kit (Akoya Biosciences, Marlborough, MA, USA) was used for IHC staining. First, the slices were baked in a constant temperature oven at 60–65 °C, dewaxed, hydrated and blocked for endogenous peroxidase activity. The antigen was repaired in AR6 (Perkin Elmer, pH = 6.0) or ethylenediaminetetraacetic acid (EDTA, pH = 9.0) antigen repair solution in a microwave oven, and then the sections were blocked in blocking buffer for 10 min and incubated with the primary antibody for 1 h. The sections were incubated with polymer HRP-conjugated secondary antibody for 10 min, and then stained with fluorophore-4 tyramine signal amplification (TSA) dye. After detecting the primary antibody, the paraffin sections were processed in AR6 or EDTA antigen repair solution in a microwave oven to remove all the primary and secondary antibodies. Then the primary antibody was applied in turn, incubated with the secondary antibody and treated with TSA. This process was repeated several times so that each antigen was labeled with a different fluorophore. Finally, the slides were stained with DAPI for 5 min and mounted with an anti-fluorescence quencher after elution. This study was divided into two multiple antibody combinations (Fig. 1B, C); Panel 1: CD8 (Abcam, ab17147, 1:100); Foxp3 (Abcam, ab22510, 1:200); Ki67 (Abcam, ab16667, 1:200); CD68 (Abcam, ab16667, 1:200); ab213363, 1:300); CD163 (Abcam, ab182422, 1:300); PD-L1 (CST, E1L3N, 1:200); Panel 2: CD66b (Abcam, ab197678, 1:100); CD20 (Abcam, ab78237, 1:100); CD11c (Abcam, ab52632, 1:500); CD56 (Abcam, ab75813, 1:500); VEGFR-2 (CST, 55B11, 1:500); IFN-γ (Bioss, bs-481R, 1:100).

### Imaging acquisition and analysis

The PerkinElmer Vectra 3 Imaging system was used to perform multispectral imaging, spectral separation, cell colocalization and segmentation imaging analysis on seven-color multistained slides. Filter blocks for multispectral imaging included 4′,6-diamidino-2- phenylindole (DAPI), fluorescein isothiocyanate (FITC), Cy3, Texas Red and Cy5. Then, we performed a full scan at 10 × and selected 3–5 areas at 20 × for a partial magnification scan (200 × final magnification) (Fig. 1A left and middle). Then we exported the images to the quantitative pathology imaging system software Inform V.2.4 (PerkinElmer) for multispectral image analysis. After automatic fluorescence background was removed, tissue segmentation, cell segmentation, and signal threshold processing are performed on 3 regions: tumor center (TC), tumor invasive front (less than 150 µm from the tumor center, TF) and peritumor (more than or equal to 150 µm from the tumor center, PT) (Fig. 1A right). On this basis, all immune cell infiltrations are counted as cell count/mm^2^ of regional tissue area (density) [23, 24].

### Statistical analysis

The nonparametric Wilcoxon rank sum test (also known as the Wilcoxon-Man-Whitney U assay) was used to compare the expression of immune biomarkers between primary tumors and liver metastases, and to compare different subgroups. All statistics were analyzed using IBM SPSS (version 26) and R software two-tailed tests. The imaging and statistical tools attached to Inform V.2.4 were used for analysis. *p* < 0.05 was considered statistically significant.

## Results

### The relationship between the density of immune cells and the clinical features of metastatic liver tumors

To investigate the correlation between immune cell biomarkers and clinical features in patients with liver metastases from colorectal cancer, we divided the patients into two groups according to the diameter and number of liver metastases. Interestingly, we found that in the comparison of the number of liver metastases, the expression of PD-L1in the primary tumor was higher in patients with metastasis = 1 than in those with metastases > 1 (*p* < 0.05). In contrast, CD66b and Foxp3 expression was higher in patients with metastases > 1 (*p* < 0.05). In liver metastases, Foxp3 was highly expressed in patients with metastases > 1 (*p* < 0.05), and there was no statistically significant difference in the expression of other cells. According to the maximum diameter of liver metastases of 3 cm (median of the largest diameter of liver metastases), we compared the biomarkers in the primary tumor and liver metastases of the two groups respectively. We found that in patients with liver metastases ≥ 3 cm, the expression of Foxp3 was higher than that in patients with liver metastases < 3 cm, in both primary and metastatic tissues respectively (*p* < 0.05). There was no significant difference in other immune cells (Table 2). In the patients with the number of liver metastases > 1, the decrease of PD-L1 and the increase of regulatory T cells (Foxp3) both suggested enhanced immunosuppression in the tumor microenvironment [10, 19]. In the patients with the maximum diameter of liver metastases ≥ 3 cm, the high expression of regulatory T cells predicted a suppressive immune microenvironment. Therefore, these data may indicate that the number of liver metastases > 1 or that the larger the maximum diameter of liver metastases is, the greater the degree of malignancy.

**Table 2:** Relationship between the density of immune cells and the clinical features of metastatic liver tumors

	Number of liver metastases				*p* value	Diameter of the metastatic tumor				*p* value
	1		> 1			< 3 cm		≥ 3 cm		
	Mean ± SD	Median	Mean ± SD	Median		Mean ± SD	Median	Mean ± SD	Median	
Primary tumor										
CD8	1.46 ± 1.35	1.03	1.90 ± 5.96	0.66	0.947	0.62 ± 0.76	0.34	0.69 ± 0.54	0.55	0.227
CD68	9.66 ± 5.68	8.44	8.38 ± 4.25	9.18	0.528	3.98 ± 2.91	2.71	4.47 ± 2.26	4.58	0.326
PD-L1	4.66 ± 3.84	3.75	3.19 ± 3.50	1.88	**0.044**	1.94 ± 2.12	1.17	1.90 ± 1.78	1.78	0.610
Ki67	6.27 ± 6.17	4.61	3.22 ± 3.28	2.10	0.096	2.15 ± 2.60	0.99	3.14 ± 3.22	2.14	0.220
Foxp3	0.68 ± 1.32	0.28	1.08 ± 1.27	0.49	**0.049**	0.22 ± 0.36	0.14	0.53 ± 0.67	0.21	**0.029**
CD163	4.35 ± 2.46	3.96	3.41 ± 1.87	3.12	0.117	1.54 ± 1.03	1.31	1.88 ± 1.00	1.67	0.206
INF-γ	0.31 ± 0.88	0.04	0.60 ± 1.17	0.02	0.481	0.21 ± 0.42	0.03	0.16 ± 0.37	0.00	0.183
CD20	0.64 ± 1.08	0.16	1.23 ± 1.59	0.62	0.063	0.26 ± 0.32	0.13	0.40 ± 0.62	0.15	0.345
CD66b	12.07 ± 15.07	6.48	13.82 ± 10.28	11.62	**0.031**	4.31 ± 2.54	4.51	4.04 ± 3.00	3.49	0.396
CD56	5.40 ± 6.18	3.28	5.43 ± 3.31	5.61	0.108	2.46 ± 1.35	2.43	1.71 ± 1.43	1.42	0.076
VEGFR-2	0.65 ± 0.61	0.53	0.74 ± 0.93	0.31	0.485	0.25 ± 0.16	0.26	0.29 ± 0.37	0.14	0.406
CD11c	0.45 ± 0.63	0.24	0.51 ± 0.82	0.29	0.605	0.16 ± 0.14	0.12	0.20 ± 0.27	0.09	0.925
Liver metastases										
CD8	063 ± 0.56	0.49	0.70 ± 0.77	0.31	0.310	0.76 ± 0.70	0.58	1.12 ± 1.61	0.53	0.806
CD68	4.43 ± 2.83	3.69	3.95 ± 2.18	4.24	0.863	3.92 ± 2.83	3.62	3.90 ± 2.76	3.54	0.940
PD-L1	2.19 ± 1.95	1.84	1.54 ± 1.88	0.95	0.924	2.15 ± 1.65	1.55	2.52 ± 2.71	1.58	0.985
Ki67	3.39 ± 3.39	2.11	1.65 ± 1.83	1.11	0.467	6.24 ± 9.26	1.26	8.02 ± 9.26	4.83	0.187
Foxp3	0.32 ± 0.56	0.12	0.47 ± 0.57	0.21	**0.003**	0.37 ± 0.28	0.37	0.76 ± 0.61	0.60	**0.011**
CD163	1.89 ± 1.07	1.76	1.47 ± 0.92	1.24	0.368	2.46 ± 1.89	2.09	2.94 ± 2.81	2.00	0.880
INF-γ	0.10 ± 0.18	0.02	0.31 ± 0.56	0.01	0.977	0.35 ± 0.51	0.18	0.32 ± 0.70	0.05	0.290
CD20	0.24 ± 0.39	0.11	0.46 ± 0.62	0.33	0.061	0.41 ± 0.74	0.09	0.42 ± 1.31	0.09	0.417
CD66b	3.56 ± 2.96	2.79	5.03 ± 2.28	5.13	0.056	2.49 ± 1.36	2.17	2.85 ± 2.70	1.61	0.664
CD56	1.83 ± 1.50	1.68	2.39 ± 1.29	2.51	0.774	0.68 ± 0.65	0.49	1.10 ± 1.59	0.46	0.685
VEGFR-2	0.26 ± 0.20	0.24	0.29 ± 0.39	0.10	0.503	0.35 ± 0.38	0.23	0.54 ± 0.85	0.17	0.720
CD11c	0.18 ± 0.23	0.11	0.18 ± 0.20	0.13	0.479	0.51 ± 0.73	0.06	0.43 ± 0.67	0.16	0.385

*p* values were obtained from Wilcoxon’s signed rank test

The bold values indicates significance at *p* < 0.05

### Comparison of immune cells between primary colon cancer and liver metastasis

**Fig. 2:**
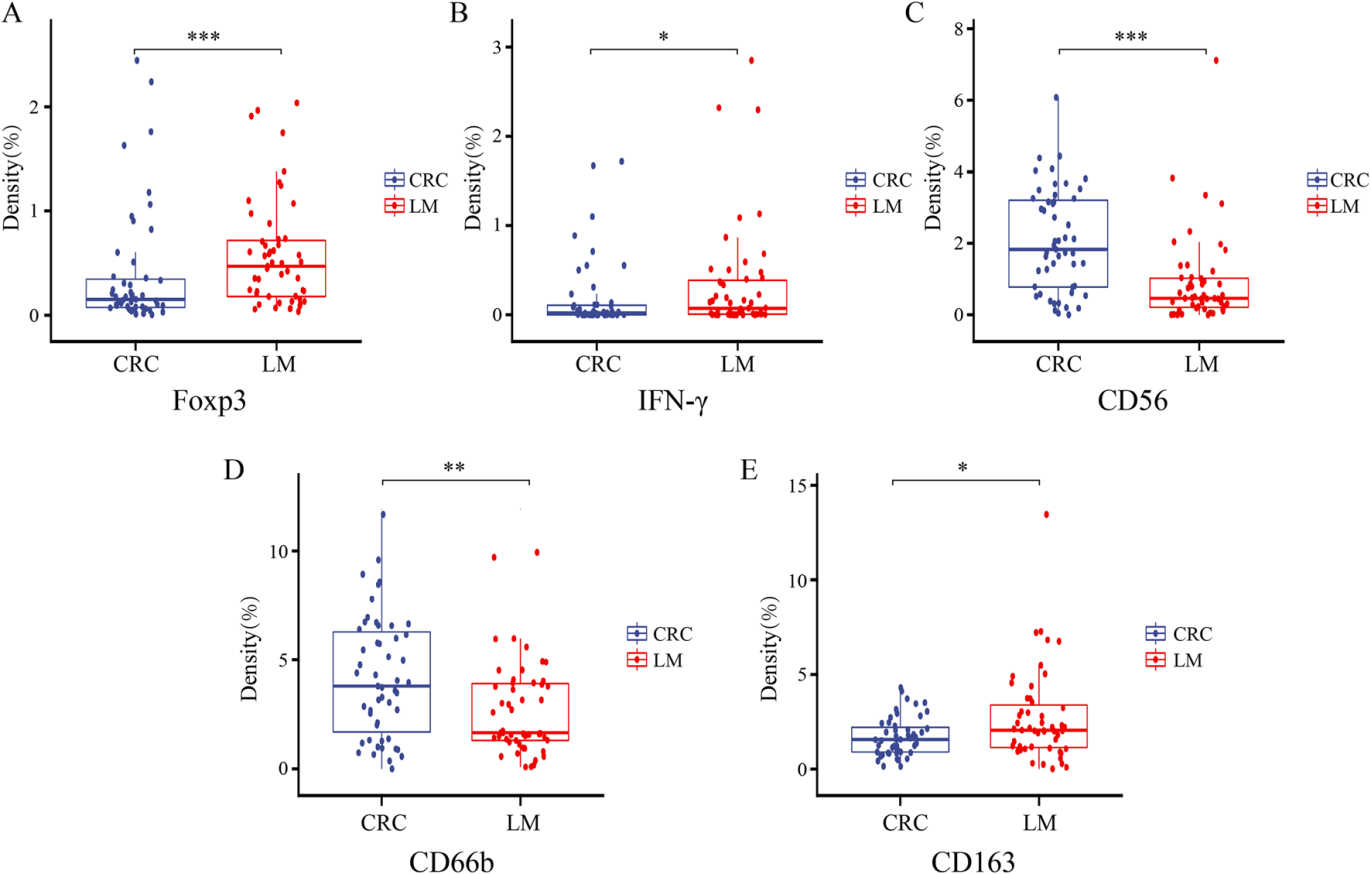
General comparison of immune markers between primary tumors and liver metastases (**A** Foxp3, **B** IFN-γ, **C** CD56, **D** CD66b, **E** CD163). (*p < 0.05, **p < 0.01, ***p < 0.001)

To understand the correlation between primary colon cancer and liver metastases in different cells of the tumor microenvironment, we used Inform software to compare the nonregional classification of primary tumors and liver metastases (including TC and TF). We found that tumor-associated neutrophils (CD66b) and NK cells (CD56) were more commonly found in primary tumors than in liver metastases, while tumor-associated regulatory T cells (Foxp3), macrophages (CD163) and IFN-γ were more commonly found in liver metastases (Fig. 2, *p* < 0.05). There was no significant difference in other T lymphocytes, B lymphocytes, dendritic cells, PD-L1, cell proliferation ability or tumor angiogenesis (Additional file 1: Fig. S1). Overall, tumor-associated T cells, B cells and dendritic cells have a similar immune status in primary tumors and liver metastases, but the expression levels of tumor-associated neutrophils, natural killer cells, regulatory T cells, macrophages and IFN-γ were different (Fig. 2 and Additional file 2: Table S1). These results suggested that the tumor microenvironment of liver metastases and primary tumors is different in the expression of certain immune cells.

### Comparison of the expression of multiple immune markers in the TC, TF and PT

**Fig. 3:**
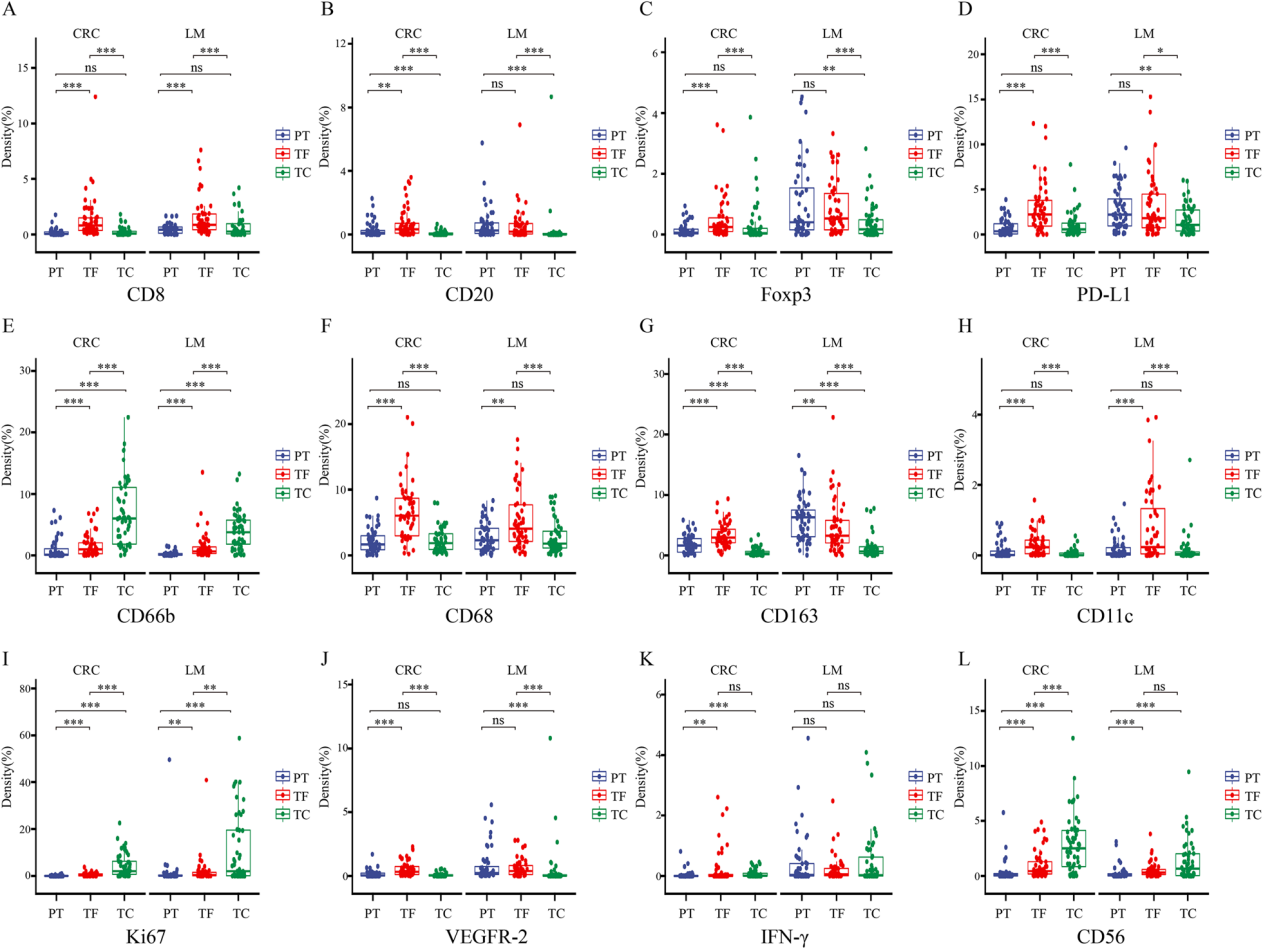
Comparison of the immune markers of the TC, TF and PT regions (**A** CD8, **B** CD20, **C** Foxp3, **D** PD-L1, **E** CD66b, **F** CD68, **G** CD163, **H** CD11c, **I** Ki67, **J** VEGFR-2, **K** IFN-γ, **I** CD56). (*p < 0.05, **p < 0.01, ***p < 0.001, *p* ≥ 0.05, not significant)

To further understand the spatial distribution of the immune microenvironment between the primary tumor and liver metastases, we divided the tumor into 3 regions, the tumor center, tumor invasive front (distance from the tumor < 150 µm) and peritumor (distance from the tumor ≥ 150 µm), and performed a comparative analysis (Additional file 3: Fig. S2 and Additional file 4: Fig. S3). First, we compared the internal areas of the primary tumor and liver metastases, found that 8 markers (CD8, Foxp3, CD68, CD163, CD20, CD11c, VEGFR-2, PD-L1) had higher expression in TF than TC region in both primary tumor and liver metastases (*p* < 0.05). In contrast, CD66b and Ki67 expression was higher in the TC than in the TF region between the primary tumor and liver metastases. Regarding the last two markers, IFN-γ showed no difference in the TC and TF regions between primary tumor and liver metastases, while CD56 had a TC greater than the TF region in the primary tumor, but there was no difference in liver metastases. In addition, the markers in the PT region showed some differences between the primary tumor and liver metastases. In the primary tumor, the expression of all 12 markers (CD8, CD68, Foxp3, CD11c, CD163, CD66b, CD56, CD20, PD-L1, VEGFR-2, Ki67, IFN-γ) in the PT region was lower than that in the TF region (*p* < 0.05). However, in the liver metastases, there was no significant difference in these 5 markers (CD20, Foxp3, VEGFR-2, PD-L1, INF-γ) in the PT and TF regions. The other 6 markers (CD8, CD68, CD66b, CD56, CD11c, Ki67) were expressed at lower levels in the PT region than in the TF region (*p* < 0.05). Interestingly, only the expression of CD163 was higher in the PT region than in the TF region (Fig. 3 and Additional file 5: Table S2). These data collectively suggested that the immune correlation between the tumor center and infiltration margin is similar in primary tumors and liver metastases, which is consistent with previous reports [25]. However, the expression levels of primary tumors and liver metastases in the infiltration edge and the peritumor region were not the same, especially CD163, which had a higher expression in the PT region.

**Fig. 4:**
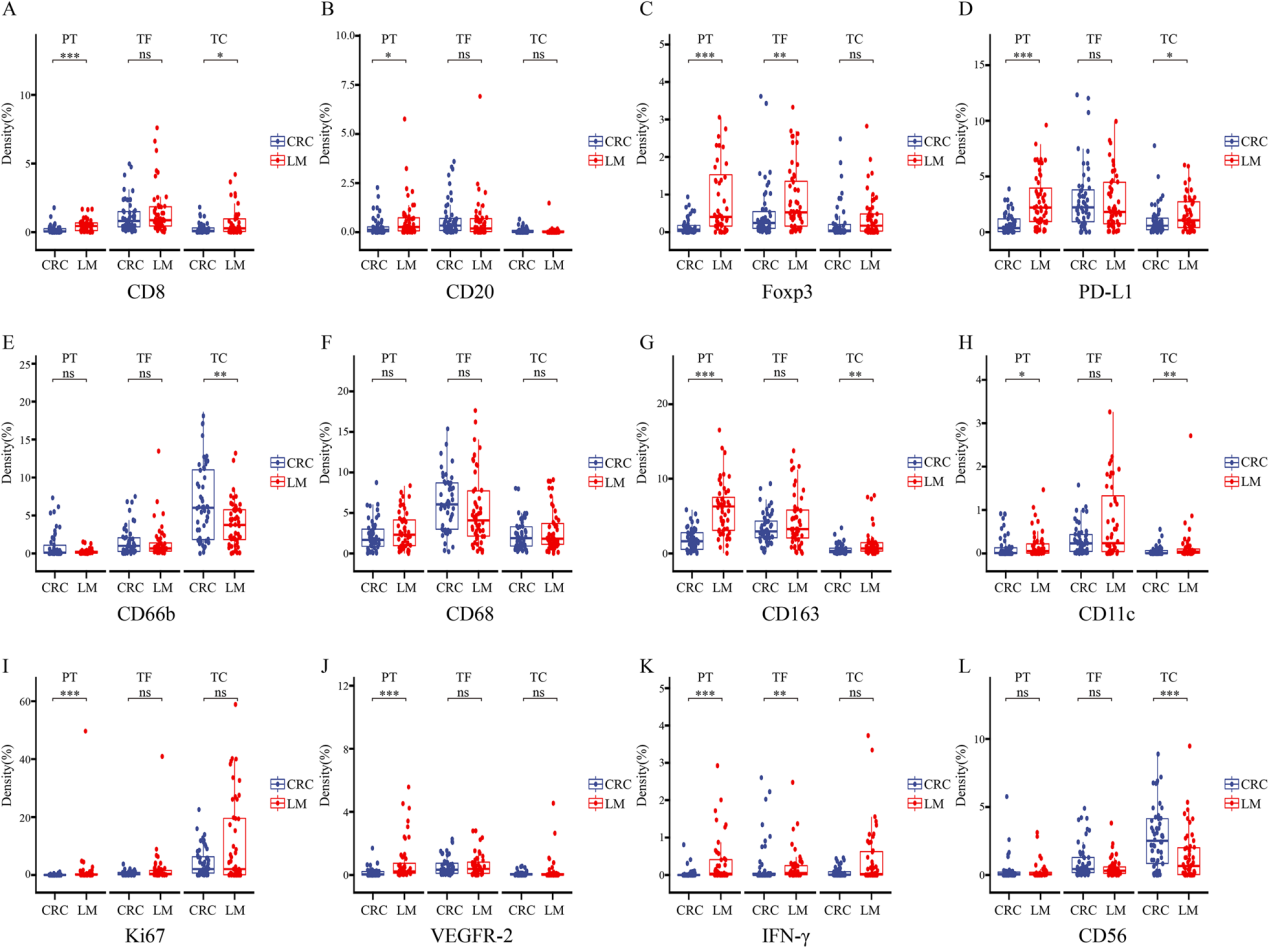
Comparison of primary tumors and liver metastases in different regions. (**A** CD8, **B** CD20, **C** Foxp3, **D** PD-L1, **E** CD66b, **F** CD68, **G** CD163, **H** CD11c, **I** Ki67, **J** VEGFR-2, **K** IFN-γ, **I** CD56). (*p < 0.05, **p < 0.01, ***p < 0.001, *p* ≥ 0.05, not significant)

The next section of the research was concerned with the comparison of primary tumors and liver metastases in different regions. In the TC region, the expression of the 4 markers (CD8, CD11c, CD163 and PD-L1) in liver metastases was significantly higher than that in the primary tumors (*p* < 0.05), but CD66b and CD56 were expressed at much higher levels in primary tumors than in liver metastases. In the TF region, only Foxp3 and IFN-γ showed higher expression in liver metastases than in primary tumors (*p* < 0.05). The other 10 markers (CD8, CD68, PD-L1, Ki67, CD163, CD20, CD11c, CD66b, CD56 and VEGFR-2) showed no difference between liver metastases and the primary tumor. In the PT region, the expression of 9 markers (CD8, PD-L1, Ki67, CD163, Foxp3, INF-γ, CD20, CD11c and VEGFR-2) was higher in liver metastases than in primary tumors (*p* < 0.05), while CD68, CD66b and CD56 expression was not different between metastatic and primary tumors (Fig. 4 and Additional file 6: Table S3). The results indicate that from the perspective of the spatial distribution of immune cells, the expression of immune cells in liver metastases is higher than that in the primary tumor in the PT region, while the expression of immune cells in liver metastases is largely similar to that of the primary tumor in the TF region. Then, in the TC region, the immunosuppression of liver metastases is significantly increased. Although CD8 expression is higher than that of the primary tumor, the literature reported that antigen-specific CD8^+^ T cells in liver metastases specifically accumulate in the liver and then be cleared or the CD8^+^ T cells may be exhausted CD8 T cells, which are a distinct cell lineage with persistent expression of inhibitory receptors and loss of effector function [26, 27]. In addition, tumor-associated macrophages are the most highly expressed immune cells in liver metastases.

### Composition of CD68^+^ and CD163^+^ cells in liver metastases and primary tumors

**Fig. 5:**
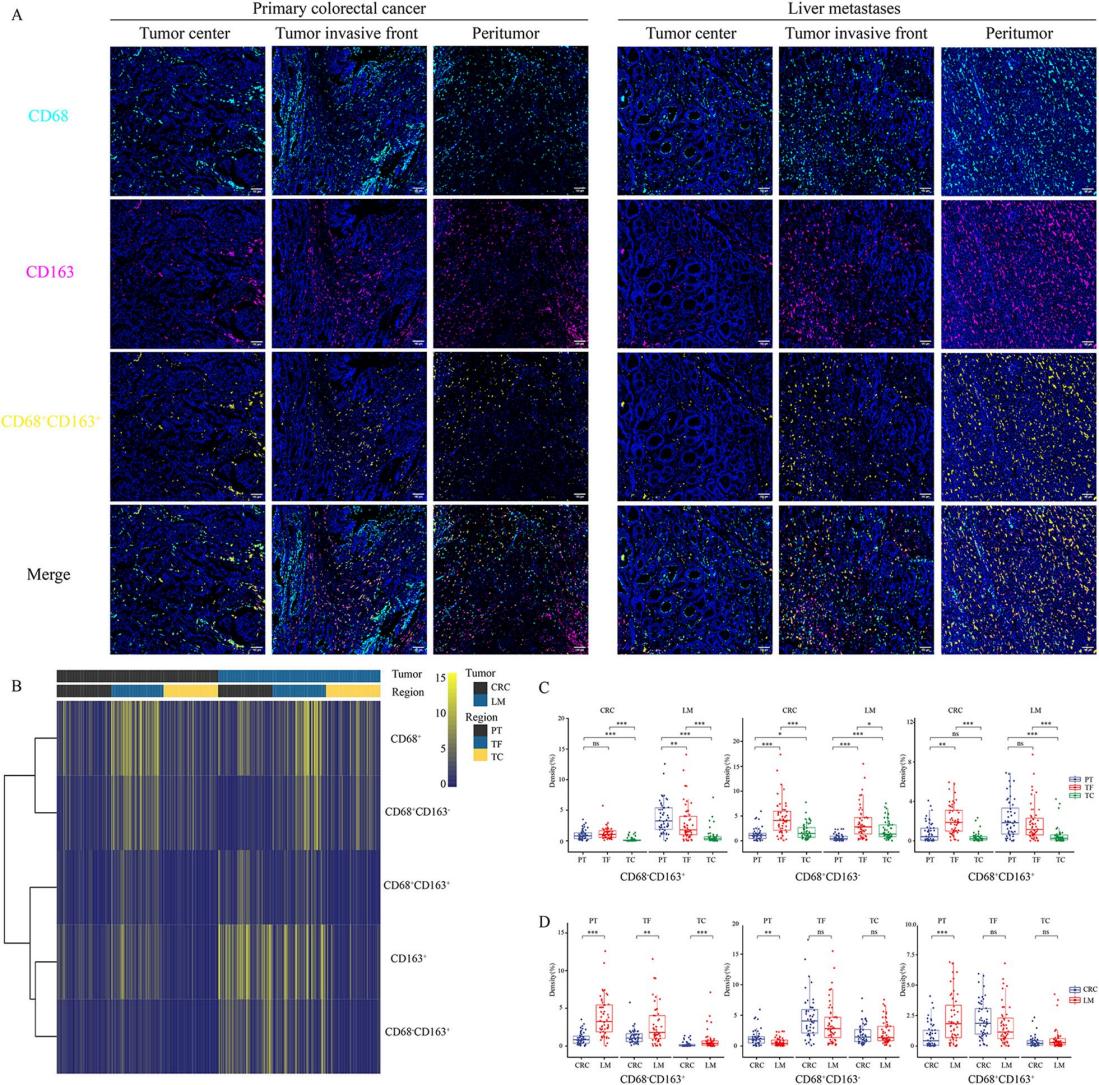
The composition of CD68^+^ and CD163^+^ cells in the liver metastases and primary tumor (**A** Typical colocalization distribution image of CD68 and CD163 in TC, TF, PT region; **B** Heatmap of the distribution of different types of macrophages based on CD68 and CD163; **C** Comparison of the internal area of the tumor in TC, TF, PT (CD68^+^CD163^+^, CD68^+^CD163^−^, CD68^−^CD163^+^); **D** Comparison of macrophage classification between primary tumor and liver metastasis (CD68^+^CD163^+^, CD68^+^CD163^−^, CD68^−^CD163.^+^). (*p < 0.05, **p < 0.01, ***p < 0.001, *p* ≥ 0.05, not significant)

To further understand the spatial distribution of TAMs between primary tumors and liver metastases, we used a colocation method based on CD68 and CD163 to compare different types of macrophages. We found that the macrophages concentrated in the PT region in the liver metastasis were mainly CD68^+^ CD163^+^ and CD68^−^CD163^+^cells. In addition, the CD68^−^CD163^+^ cells in the PT, TF, and TC regions were all expressed at higher levels in liver metastases than in primary tumors. Moreover, CD68^+^CD163^−^ staining showed that the primary tumor was larger than the liver metastases in the PT region, and there was no significant difference in the TF and TC regions (Fig. 5 and Additional file 7: Fig. S4). In summary, tumor-associated macrophages are spatially rearranged in liver metastases, and CD68^−^CD163^+^ macrophages constitute a major proportion in liver metastases. CD68 serves as a pan-macrophage marker for tumor-associated macrophages, and CD163-labeled macrophages are mainly M2 macrophages, which promote tumor growth and metastasis [28, 29]. The macrophages were further divided by the status of the two markers, and it could be seen that there were significant differences between the liver metastases and the primary tumor, especially CD68^−^CD163^+^ macrophages, which may play a key role in the immunosuppression of the liver metastases microenvironment.

### Comparison of the three immunotypes according to the degree of CD8 infiltration

**Fig. 6:**
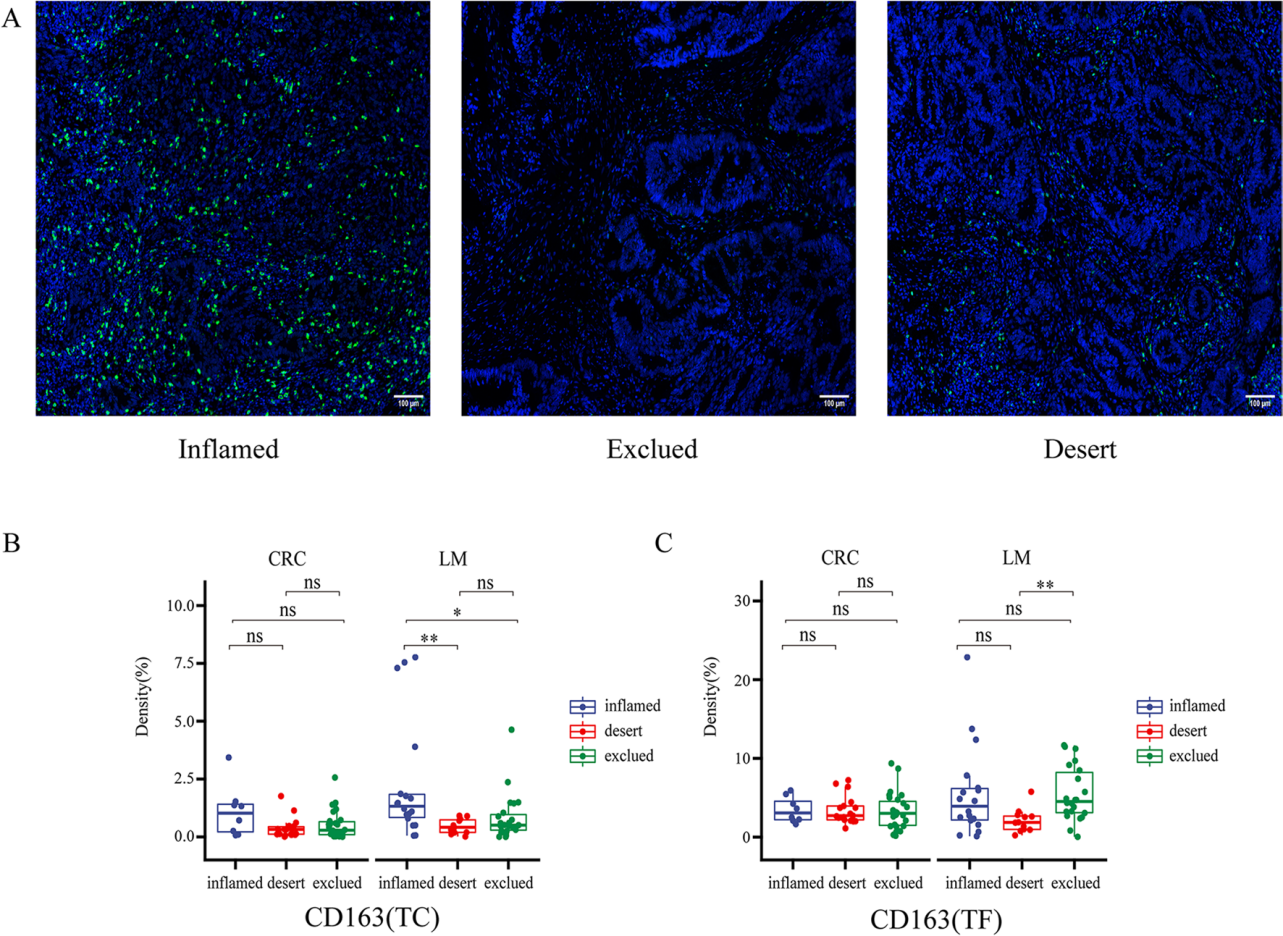
Comparison of the three immunotypes according to the degree of CD8 infiltration. **A** Immunofluorescence images of three immunophenotypes (immune-inflamed, immune desert, immune-excluded) **B** Comparison of the three immunotypes at the front of the tumor (CD163). **C** Comparison of the three immunotypes at the center of the tumor (CD163). (*p < 0.05, **p < 0.01, *p* ≥ 0.05, not significant)

To better understand the immune status of patients with liver metastases from colon cancer, we classified the tissues into 3 types according to the degree of CD8 T-cell infiltration: immune-inflamed, immune desert, and immune-excluded [30]. Then, in the liver metastasis a comparative analysis of CD68, CD163, Foxp3, PD-L1, and Ki67 on the same staining panel as CD8 T cells showed that CD163 was significantly increased in the immune rejection phenotype observed in the TF region, and in the TC region, CD163 was significantly elevated in immunoinflammatory tumors. However, there was no significant difference in the 3 types of immune cells in the primary tumors (Fig. 6). Overall, these results further illustrate that CD163 may play an important role in liver immunosuppression.

## Discussion

In recent years, multiplex immunofluorescence has been demonstrated in several studies as a very valuable and meaningful tool for the immunoassay of tumor tissues [31, 32]. Compared to previous studies, we utilized the multiplex immunofluorescence analysis to visualize the expression of multiple immune cells on a single slide, which would give us a comprehensive overview of the tumor microenvironment and avoid the spatial heterogeneity compared with conventional IHC on consecutive slides. In this study, we used PerkinElmer Inform software to compare and analyze various aspects of primary colorectal cancer and liver metastases (T cells, B cells, macrophages, neutrophils, DCs, NK cells, lymphokines, PD-L1, tumor angiogenesis and cell proliferation). Previous research has mainly focused on T lymphocyte [8, 33, 34], but T cells are insufficient for representing the complex microenvironment of the immune system. Macrophages, B cells, neutrophils, etc., also play an important role in the tumor microenvironment [35–38]. Therefore, the multicellular perspective allows us to better understand the tumor microenvironment between primary tumors and liver metastases. In addition, according to recent reports, the immune microenvironment of tumors mainly depends on two regions (TC and TF), but the definition of the TF region is not clear. In our research and observation, the spatial distribution of immune cells at the edge of tumor invasion was different. At a distance of less than 150 µm from the edge of the tumor, immune cells obviously clump together, similar to the formation of an “immune band”. At the same time, in the area more than or equal to 150 µm from the tumor, some immune cells also showed an increase, illustrating that the tumor microenvironment is dynamic. Therefore, it is necessary for us to conduct a more detailed spatial analysis of the tumor infiltration margin to better analyze the microenvironment and strengthen our understanding of the spatial distribution of the microenvironment.

In our research, we found that some immune cells have a certain correlation with the number and size of liver metastases. One possible explanation for this might be that the immunosuppressive effect of the tumor is stronger when the number of liver metastases is > 1 or the diameter is ≥ 3 cm, or it may only be due to individual differences in patients. In the analysis of the expression of a variety of immune cells between the primary tumor and liver metastases, it was found that, overall, the liver metastases and primary tumors have similar immune statuses in regard to the presence of T cells, B cells, dendritic cells, PD-L1, tumor angiogenesis, and cell proliferation. However, hepatic immunosuppression cannot be clearly seen from the overall differences in neutrophils, macrophages, regulatory T cells, and INF-γ in primary and liver metastases. From the perspective of spatial distribution, the immune cells of the primary tumor and liver metastases are mainly concentrated in the invasive front of the tumor, less than 150 µm from the tumor, which is much higher than the tumor center, which confirms previous research [39]. Compared with the primary tumor, liver metastases express more immune cells in the peritumor area more than or equal to 150 µm away from the tumor, especially CD163-labeled macrophages, which have the highest expression in the PT region. It is well known that CD163 can enhance the migration and invasion of colorectal cancer cells [40] and is related to the poor prognosis of patients [41]. This may be an important reason for the immunosuppression of liver metastases.

Furthermore, we conducted a colocalization analysis of CD68- and CD163-labeled macrophages, and found that the spatial distribution of macrophages changed significantly. In the PT region, the distribution of macrophages in primary tumors and liver metastases is significantly different. The primary tumors were mainly CD68^+^CD163^−^ cells, while the liver metastases were mainly CD68^+^CD163^+^ and CD68^−^CD163^+^ cells. Interestingly, CD68^−^CD163^+^ cells showed a significant increase in liver metastases in the spatial distribution among TC, TF, and PT regions compared with primary tumors. Research reports that macrophages in liver metastases can cause CD8 T-cell loss and reduce the effectiveness of immunotherapy [26] which also suggests that the high expression of CD68^−^CD163^+^ macrophages may be related to the immunosuppression of liver metastases. According to the immune status in different spatial distribution areas of the liver metastasis and the primary tumor, we speculate that CD68^−^CD163^+^ macrophages may not only inhibit or eliminate the function of CD8 T cells, but also inhibit or eliminate B cells, dendritic cells and PD-L1. Furthermore, we also found significant differences in the expression of CD163 in different cell types by immunophenotyping. This represents a difficult problem in the administration of immunotherapy for patients with liver metastases. Notably, a recent phase I clinical trial study found that the combination of regorafenib and nivolumab in MSS colorectal cancer patients reached an objective response rate of 33%. However, none of the patients with liver metastases are responsive [42], suggesting that the immunosuppressive TME in liver metastases may hamper the efficacy of PD-1 blockade. In our study, we found that CD68^+^CD163^−^ macrophages were closely related to the immunosuppression of liver metastases. In future, it is promising that specific targeting and modulation of this group of macrophages may have potential to stimulate tumor immunity and enhance anti-tumor activity of PD-1 blockade. Addressing the problem of immunosuppression of macrophages in liver metastases may lead to breakthroughs in immunotherapy. To our knowledge, there is few studies to analyze the similarities and differences in the microenvironment between liver metastases and the primary tumor by using multiple types of cells from a pathological perspective.

However, our research has some limitations. First, this was a retrospective study. In addition, the sample size was small, and the selection of various immune cell markers was not sufficient. It does not fully represent all types of immune cells. The spatial extent of a PT region larger than 150 µm is not precisely defined, and not all tumors have a PT region. When the tumor is observed to be more poorly differentiated, we can only divide it into TF and TC regions, and cannot clearly distinguish the PT region. Therefore, more studies are needed in the future to confirm our results.

## Conclusion

In primary colorectal cancer and liver metastases, the tumor microenvironment is complex and dynamic, and the various types of immune cells in the microenvironment also show differences in their spatial distribution. Among them, the most significant change is in the distribution of macrophages in liver metastases, which may bring about functional changes. The high expression of CD68^−^CD163^+^ macrophages in liver metastases suggests that CD68^−^CD163^+^ may be related to intrahepatic immunosuppression and weak immunotherapy effects.

## Supplementary Information

**Additional file 1: Figure S1.** General comparison of immune markers between primary tumors and liver metastases (A: CD8, B: CD20, C: CD68, D: CD11c, E: VEGFR-2, F: PD-L1, G: Ki67) (p ≥ 0.05, not significant).

**Additional file 2: Table S1.** General comparison of immune markers between primary tumors and liver metastases.

**Additional file 3: Figure S2. **Representative multiplex immunofluorescence images of 6 cell markers from panel 1 in the tumor center (TC), tumor invasive front (TF), and peritumoral (PT) regions of primary tumors and liver metastases.

**Additional file 4: Figure S3.** Representative multiplex immunofluorescence images of 6 cell markers from panel 2 in the tumor center (TC), tumor invasive front (TF), and peritumoral (PT) regions of primary tumors and liver metastases.

**Additional file 5: Table S2**. Comparison of the immune markers of the TC, TF and PT regions.

**Additional file 6: Table S3. **Comparison of primary tumors and liver metastases in different regions.

**Additional file 7: Figure S4.** Comparison of the three immunotypes according to the degree of CD8 infiltration. A: Comparison of the three immunotypes at the front of the tumor (CD68, Foxp3, PD-L1 and Ki67). C: Comparison of the three immunotypes at the center of the tumor (CD68, Foxp3, PD-L1 and Ki67). (*p< 0.05, p ≥ 0.05, not significant).

## Data Availability

The datasets generated and/or analyzed during the current study are available from the corresponding author upon reasonable request.

## Abbreviations

TC: Tumor center
TF: Tumor invasive front
PT: Peritumor
CRC: Colorectal cancer
LM: Liver metastases
MSI-H: High microsatellite instability
MMR-D: Mismatch repair deficiency
TAMs: Tumor-associated macrophages
DCs: Dendritic cells
NK cells: Natural killer cells
EDTA: Ethylenediaminetetraacetic acid
TSA: Tyramine signal amplification
DAPI: 4',6-Diamidino-2- phenylindole
FITC: Fluorescein isothiocyanate
