# Supplementary material for: Multi-perspective comparison of the immune microenvironment of primary colorectal cancer and liver metastases
Source: J Transl Med. 2022 Oct 4;20:454. doi: 10.1186/s12967-022-03667-2 (PMC9533561; doi:10.1186/s12967-022-03667-2)
Supplement: Supplementary file 1 — Additional file 1: Figure S1. General comparison of immune markers between primary tumors and liver metastases (A: CD8, B: CD20, C: CD68, D: CD11c, E: VEGFR-2, F: PD-L1, G: Ki67) (p ≥ 0.05, not significant). [file 12967_2022_3667_MOESM1_ESM.pdf]

Supplementary Material

1 Supplementary Figures and Tables

1.1 Supplementary Figures

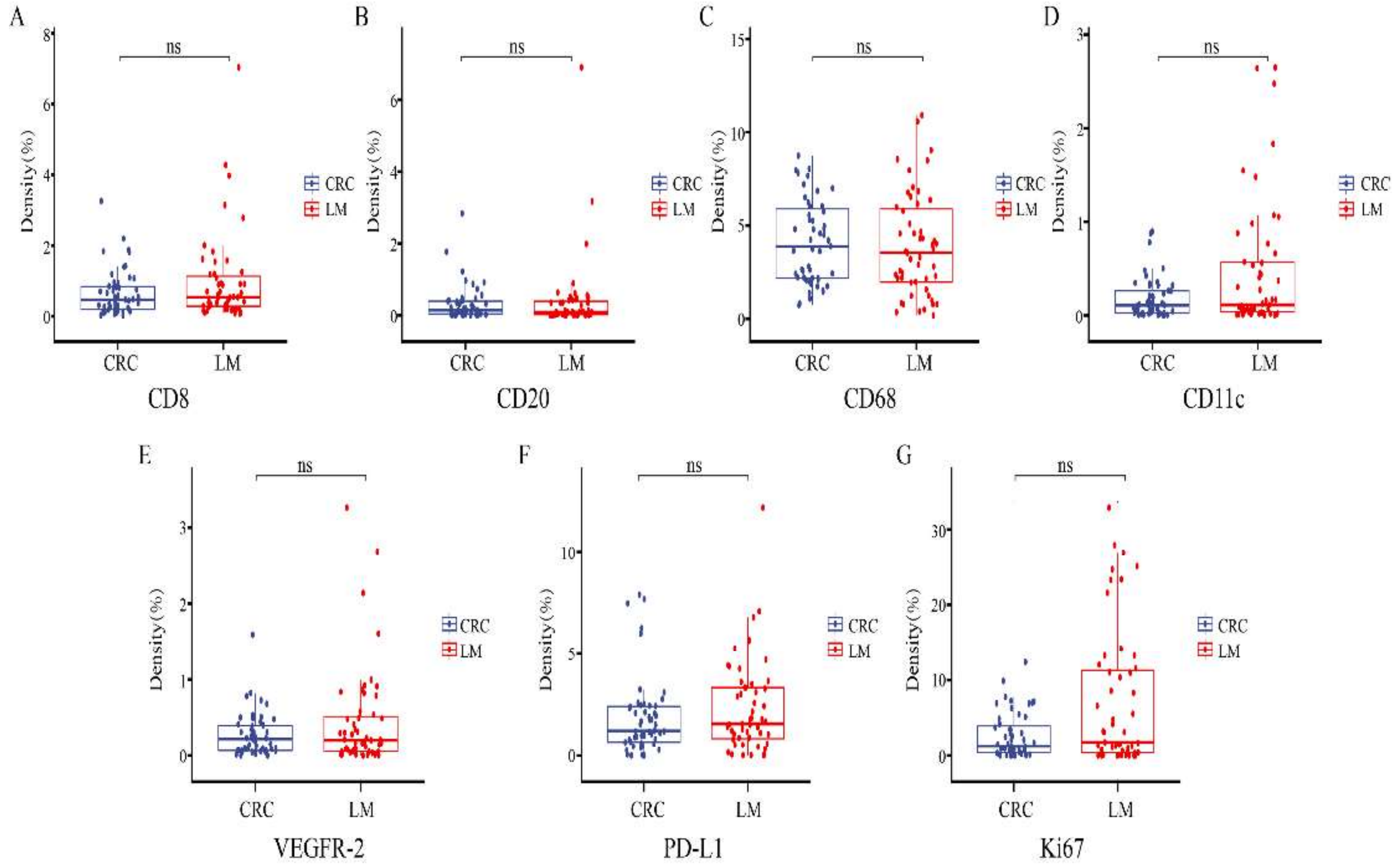

Additional file 1: Figure S1 General comparison of immune markers between primary tumors and liver metastases (A: CD8, B: CD20, C: CD68, D: CD11c, E: VEGFR-2, F: PD-L1, G: Ki67) ($p \geqslant 0.05$, not significant)
