# Supplementary material for: Multi-perspective comparison of the immune microenvironment of primary colorectal cancer and liver metastases
Source: J Transl Med. 2022 Oct 4;20:454. doi: 10.1186/s12967-022-03667-2 (PMC9533561; doi:10.1186/s12967-022-03667-2)
Supplement: Supplementary file 2 — Additional file 2: Table S1. General comparison of immune markers between primary tumors and liver metastases. [file 12967_2022_3667_MOESM2_ESM.pdf]

1.2 Supplementary Tables

Additional file 2: Table S1 General comparison of immune markers between primary tumors and liver metastases

	Primary tumor			Liver metastases			*p* value
	Mean±SD	Median	25%, 75%	Mean±SD	Median	25%, 75%	
CD8	0.66±0.65	0.47	0.19 0.85	0.99±1.27	0.54	0.28 1.18	0.210
CD68	4.24±2.57	3.89	2.17 6.03	3.91±2.76	3.54	1.94 5.99	0.416
PD-L1	1.92±1.93	1.24	0.67 2.44	2.35±2.26	1.58	0.82 3.35	0.286
Ki67	2.67±2.96	1.28	0.41 4.19	7.18±9.21	1.81	0.36 11.56	0.136
Foxp3	0.38±0.56	0.15	0.07 0.36	0.58±0.52	0.47	0.18 0.73	**< 0.001**
CD163	1.72±1.02	1.56	0.90 2.28	2.72±2.41	2.06	1.11 3.53	**0.025**
INF-γ	0.18±0.39	0.02	0.00 0.11	0.34±0.61	0.07	0.01 0.40	**0.030**
CD20	0.33±0.50	0.15	0.04 0.40	0.41±1.07	0.09	0.03 0.42	0.292
CD66b	4.16±2.77	3.79	1.38 6.39	2.68±2.17	1.66	1.30 3.94	**0.008**
CD56	2.06±1.43	1.83	0.77 3.25	0.90±1.25	0.46	0.19 1.03	**< 0.001**
VEGFR-2	0.27±0.29	0.22	0.07 0.40	0.45±0.67	0.21	0.05 0.54	0.647
CD11c	0.18±0.22	0.11	0.02 0.27	0.46±0.69	0.11	0.04 0.57	0.159

p values were obtained from Wilcoxon's signed rank test

The bold values indicates significance at p<0.05
