# Supplementary material for: Multi-perspective comparison of the immune microenvironment of primary colorectal cancer and liver metastases
Source: J Transl Med. 2022 Oct 4;20:454. doi: 10.1186/s12967-022-03667-2 (PMC9533561; doi:10.1186/s12967-022-03667-2)
Supplement: Supplementary file 3 — Additional file 3: Figure S2. Representative multiplex immunofluorescence images of 6 cell markers from panel 1 in the tumor center (TC), tumor invasive front (TF), and peritumoral (PT) regions of primary tumors and liver metastases. [file 12967_2022_3667_MOESM3_ESM.pdf]

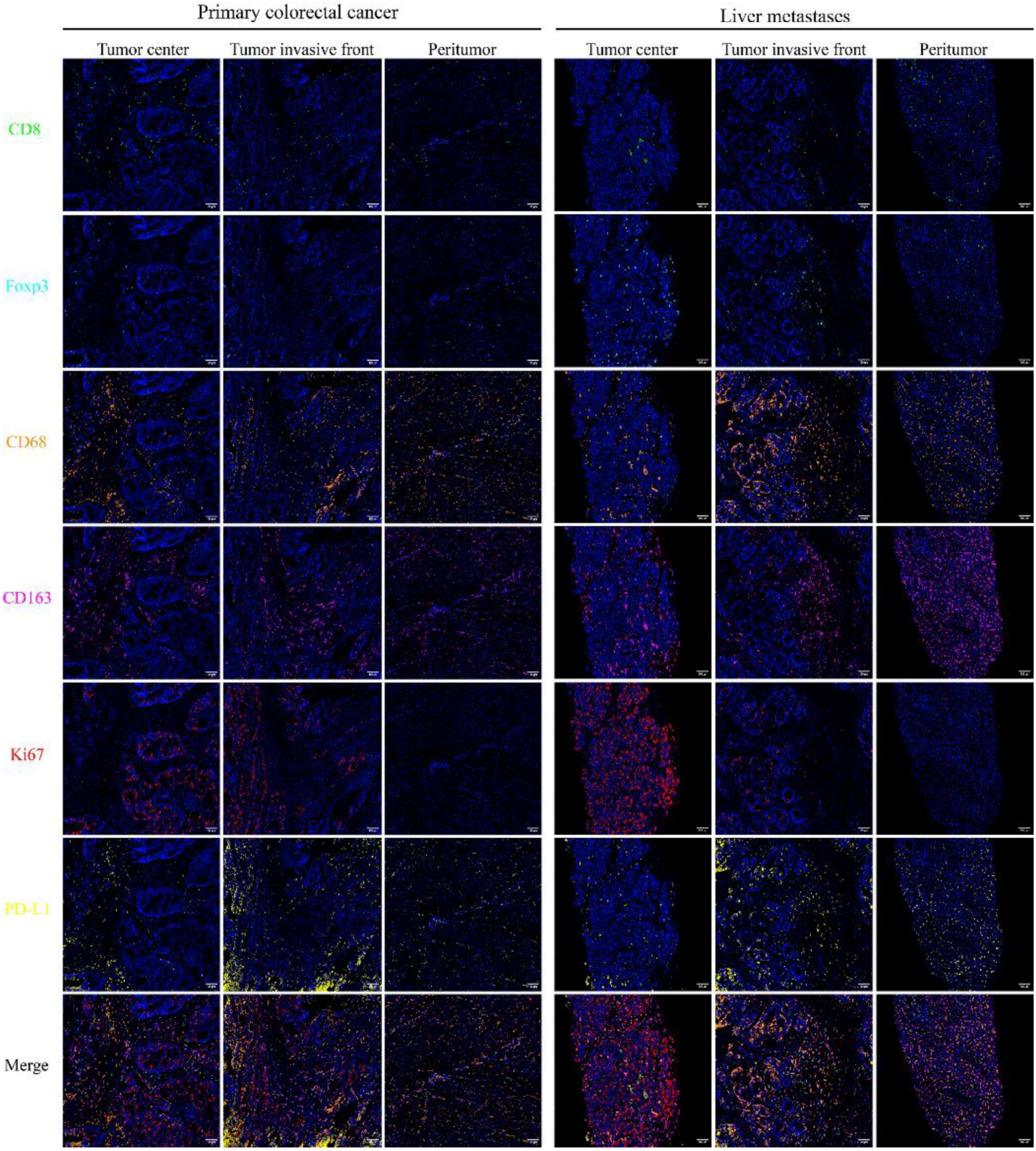

Additional file 3: Figure S2 Representative multiplex immunofluorescence images of 6 cell markers from panel 1 in the tumor center (TC), tumor invasive front (TF), and peritumoral (PT) regions of primary tumors and liver metastases
