# Supplementary material for: Multi-perspective comparison of the immune microenvironment of primary colorectal cancer and liver metastases
Source: J Transl Med. 2022 Oct 4;20:454. doi: 10.1186/s12967-022-03667-2 (PMC9533561; doi:10.1186/s12967-022-03667-2)
Supplement: Supplementary file 6 — Additional file 6: Table S3. Comparison of primary tumors and liver metastases in different regions. [file 12967_2022_3667_MOESM6_ESM.pdf]

Additional file 6: Table S3 Comparison of primary tumors and liver metastases in different regions

	Primary tumor			Liver metastases			*p* value
	Mean±SD	Median	25%, 75%	Mean±SD	Median	25%, 75%	
Tumor center							
CD8	0.25±0.38	0.09	0.03 0.31	0.67±0.97	0.27	0.05 1.01	**0.020**
CD68	2.29±1.83	1.87	0.86 3.31	2.77±2.49	1.80	1.13 3.78	0.554
PD-L1	1.05±1.38	0.58	0.20 1.35	1.62±1.56	1.06	0.37 2.77	**0.037**
Ki67	4.37±5.05	2.04	0.62 6.59	11.05±14.80	2.02	0.08 19.70	0.520
Foxp3	0.32±0.71	0.04	0.01 0.22	0.39±0.57	0.17	0.03 0.49	0.134
CD163	0.56±0.69	0.32	0.10 0.70	1.25±1.80	0.65	0.31 1.45	**0.005**
INF-γ	0.07±0.13	0.01	0.00 0.10	0.48±0.92	0.02	0.00 0.64	0.120
CD20	0.07±0.14	0.01	0.00 0.10	0.22±1.21	0.00	0.00 0.06	0.188
CD66b	6.83±5.34	6.00	1.75 11.11	3.93±2.94	3.76	1.74 5.77	**0.009**
CD56	2.91±2.61	2.50	0.83 4.15	1.40±1.85	0.67	0.03 2.02	**< 0.001**
VEGFR-2	0.08±0.13	0.04	0.00 0.09	0.44±1.64	0.02	0.00 0.09	0.517
CD11c	0.05±0.11	0.00	0.00 0.07	0.15±0.40	0.04	0.00 0.12	**0.010**
Tumor invasive front							
CD8	1.36±1.95	0.81	0.34 1.53	1.47±1.67	0.87	0.38 1.84	0.475
CD68	6.74±4.39	6.06	2.92 8.73	5.43±4.40	4.07	2.52 7.88	0.054
PD-L1	3.01±2.88	2.22	0.91 3.90	3.04±3.34	1.82	0.82 4.59	0.658
Ki67	0.57±0.71	0.29	0.07 0.90	1.99±5.82	0.36	0.07 1.86	0.147
Foxp3	0.51±0.75	0.25	0.10 0.58	0.86±0.87	0.53	0.12 1.46	**0.009**
CD163	3.38±2.01	2.94	2.08 4.40	4.70±4.31	3.24	1.84 5.72	0.298
INF-γ	0.25±0.59	0.00	0.00 0.06	0.22±0.43	0.05	0.02 0.30	**0.008**
CD20	0.69±0.93	0.33	0.07 0.75	0.56±1.07	0.18	0.04 0.74	0.170
CD66b	1.60±1.88	1.00	0.24 2.10	1.41±2.21	0.65	0.35 1.72	0.594
CD56	1.00±1.28	0.44	0.17 1.32	0.52±0.70	0.30	0.16 0.69	0.064
VEGFR-2	0.54±0.58	0.33	0.11 0.74	0.60±0.70	0.38	0.12 0.98	0.930
CD11c	0.32±0.34	0.23	0.05 0.44	0.79±1.02	0.24	0.05 1.53	0.206
Peritumor							
CD8	0.19±0.31	0.06	0.02 0.26	0.47±0.42	0.44	0.08 0.60	**< 0.001**
CD68	2.17±1.88	1.66	0.82 3.09	2.87±2.22	2.32	1.32 4.94	0.103
PD-L1	0.76±0.93	0.36	0.05 1.19	2.76±2.36	2.20	1.16 5.10	**< 0.001**
Ki67	0.08±0.19	0.01	0.00 0.06	1.44±6.89	0.13	0.04 0.45	**< 0.001**
Foxp3	0.15±0.21	0.06	0.02 0.18	1.03±1.28	0.40	0.14 1.73	**< 0.001**
CD163	1.82±1.46	1.63	0.49 2.81	6.02±3.49	6.29	2.99 8.02	**< 0.001**
INF-γ	0.04±0.13	0.00	0.00 0.00	0.40±0.84	0.03	0.00 0.44	**< 0.001**
CD20	0.29±0.47	0.12	0.02 0.26	0.61±0.98	0.26	0.08 0.80	**0.011**
CD66b	0.10±1.76	0.10	0.03 1.11	0.25±0.34	0.11	0.07 0.36	0.661
CD56	0.32±0.91	0.06	0.00 0.23	0.27±0.61	0.05	0.01 0.33	0.469
VEGFR-2	0.15±0.28	0.04	0.00 0.22	0.81±1.28	0.22	0.17 1.13	**< 0.001**
CD11c	0.15±0.26	0.01	0.00 0.14	0.18±0.30	0.05	0.01 0.26	**0.022**

p values were obtained from Wilcoxon's signed rank test

The bold values indicates significance at $p<0.05$
