# Supplementary material for: Multi-perspective comparison of the immune microenvironment of primary colorectal cancer and liver metastases
Source: J Transl Med. 2022 Oct 4;20:454. doi: 10.1186/s12967-022-03667-2 (PMC9533561; doi:10.1186/s12967-022-03667-2)
Supplement: Supplementary file 7 — Additional file 7: Figure S4. Comparison of the three immunotypes according to the degree of CD8 infiltration. A: Comparison of the three immunotypes at the front of the tumor (CD68, Foxp3, PD-L1 and Ki67). C: Comparison of the three immunotypes at the center of the tumor (CD68, Foxp3, PD-L1 and Ki67). (*p< 0.05, p ≥ 0.05, not significant). [file 12967_2022_3667_MOESM7_ESM.pdf]

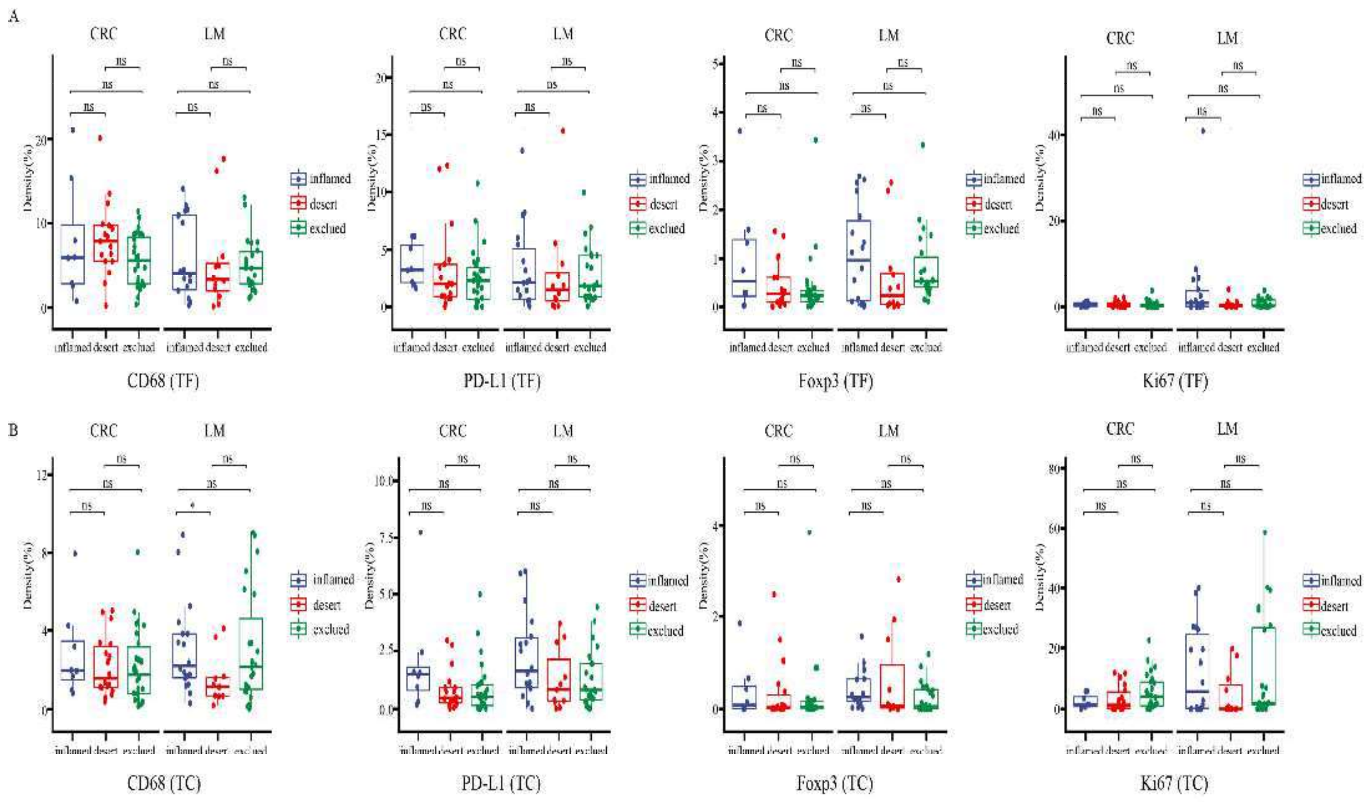

Additional file 7: Figure S4 Comparison of the three immunotypes according to the degree of CD8 infiltration. A: Comparison of the three immunotypes at the front of the tumor (CD68, Foxp3, PD-L1 and Ki67). C: Comparison of the three immunotypes at the center of the tumor (CD68, Foxp3, PD-L1 and Ki67). (*$p< 0.05$, $p \geqslant 0.05$, not significant)
